# Revolutionizing Atrial Fibrillation Treatment: The Robotic Convergent Plus Procedure

**DOI:** 10.7759/cureus.57835

**Published:** 2024-04-08

**Authors:** Zain Khalpey, Ujjawal Kumar, Usman Aslam, Yoaav Krauthammer, Rahul Doshi

**Affiliations:** 1 Department of Cardiothoracic Surgery, HonorHealth, Scottsdale, USA; 2 School of Clinical Medicine, University of Cambridge, Cambridge, GBR; 3 General Surgery Residency Program, HonorHealth, Phoenix, USA; 4 Department of Electrophysiology, HonorHealth, Scottsdale, USA

**Keywords:** electrophysiological mapping, persistent atrial fibrillation, hybrid ablation, robotic cardiac surgery, convergent procedure, atrial fibrillation (af)

## Abstract

Atrial fibrillation (AF) is widely accepted to be the most common sustained arrhythmia, with an increasing incidence over time. This is thought to be due to the aging population across the world. AF occurs when abnormal electrical foci result in disorganization of atrial depolarization, though the exact pathophysiology leading to these abnormal foci is not well understood. A range of interventions exist for AF - pharmacological therapies (anti-arrhythmic or negative chronotropic medications), cardioversion, or ablations to interrupt the abnormal conduction pathways. Ablation may be via a catheter-based approach, via a surgical approach using the “Maze” procedure (Cox-Maze IV), or more recently, via a hybrid approach. This first involves a surgical epicardial ablation, with catheter-based endocardial ablation following a few weeks later to ensure durable transmural lesion sets via the “Convergent” procedure. We describe the use of the Da Vinci Xi™ robotic platform to improve the procedure, allowing continuous and improved visualization of the anatomy without the need for potentially harmful retraction of the atrial appendage or the back of the left atrium, as well as increased precision with our mapping tools and more complete ablation. Here, we highlight the advantages over a non-robotic (subxiphoid) Convergent procedure, while outlining the key operative steps in undertaking the “Robotic Convergent Plus” procedure using the Da Vinci Xi™ robotic surgical system.

## Introduction

Atrial fibrillation (AF) is the most common sustained arrhythmia affecting around five million US adults [1]. With an aging population, its prevalence is expected to rise above 12 million by the year 2030 [2,3]. As most individuals with AF are asymptomatic and undiagnosed, the true prevalence is much higher. Though most patients with AF are often asymptomatic, the presence of this arrhythmia confers a significant morbidity and mortality burden. In 2019, 183,321 death certificates in the USA cited AF as a contributor, with 14.5% directly attributed to AF [4]. Additionally, AF contributes to the financial strain on US healthcare, accounting for $34 billion in 2021-2022, a figure expected to rise with the aging population [5].

The exact pathophysiology of AF is not fully understood. Atrial myocardial stretch, inflammation, and scarring observed in AF progress to the development of ectopic foci of electrical activity. These foci result in disorganized atrial depolarizations leading to AF. It is well established that metabolic, electrical, and mechanical remodeling of the left atrium occurs in response to the arrhythmia, further perpetuating AF.

Rapid episodes of AF are associated with turbulent blood flow, resulting in the formation of intra-cardiac thrombi that may embolize and cause a stroke. AF is associated with an approximately five-fold increase in the risk of embolic stroke [6]. Prophylactic anticoagulation is therefore fundamental in AF management to mitigate thromboembolic risk. This affects trauma care due to increased hemorrhage, especially cerebral hemorrhage, and further increases mortality in AF patients with traumatic injuries [7].

Anti-arrhythmic drugs and pulmonary vein isolation (PVI) are primary treatments for AF, targeting ectopic beats originating from the pulmonary veins. However, non-pulmonary vein structures like the left atrial posterior wall, left atrial appendage (LAA), coronary sinus, superior vena cava, crista terminalis, and ligament of Marshall also play a role. For persistent AF, isolated PVI may be less effective, making it crucial to also ablate these non-PV areas for successful treatment.

Surgical ablation methods, particularly the Cox-Maze IV (Maze) procedure, have become prominent alongside catheter-based therapies for refractory AF. A surgical approach allows the creation of a “box lesion” that connects PVI lines with roof and floor lines, which isolates the entire posterior wall. Additionally, the LAA is externally excluded, significantly reducing the risk of thromboembolic events. 80%-90% of patients do not require anti-arrhythmic medications after surgical ablation via a Maze procedure [8]. The creation of continuous linear lesions without potential pro-arrhythmic gaps continues to be a challenge in catheter-based approaches. Additionally, catheter-based endocardial-only ablations are less durable, with a recent meta-analysis reporting a 63% rate of posterior wall reconnection [9].

While the benefits of open surgical ablation are evident, the Maze procedure has several significant disadvantages. Cardiopulmonary bypass (CPB) and sternotomy are essential and are associated with higher morbidity and mortality than catheter-based ablations. Subsequent iterations of the Maze procedure, such as the “Mini-Maze,” achieve similar lesion sets without the need for a median sternotomy. The Mini-Maze can be unilateral or bilateral and does not necessarily require CPB. However, it does present significant anatomical challenges, with multiple incisions required to achieve suitable access to key structures, resulting in significant postoperative pain and potential for wound complications.

An alternative is the sequential hybrid ablation approach, like the Convergent procedure, which involves an interval endocardial radiofrequency ablation (RFA) after a surgical epicardial ablation (CryoMaze). Hybrid approaches are rapidly gaining popularity as they are less invasive and provide a more complete ablation of AF substrates. The recent SURHYB trial has shown that patients with longstanding non-paroxysmal AF undergoing this hybrid approach had a significantly lower incidence of postoperative AF or atrial tachycardia and a lower need for continued use of Class I or III anti-arrhythmic medications. Hospitalizations for cardiac complications were also significantly reduced in the hybrid group compared to those solely undergoing a CryoMaze procedure, with these two results being seen across all subgroups [8].

The hybrid approach was developed to achieve electrophysiologically validated transmural lesion sets that target additional foci of electrical activity, as well as excluding the LAA. The Convergent procedure is one such hybrid approach undertaken by a multidisciplinary team of cardiac surgeons and electrophysiologists. This treatment is for patients with persistent AF that is refractory to prior ablation(s) and involves an initial surgical epicardial ablation performed without CPB and interval catheter-based endocardial ablation performed as a sequential step (either on the same day or a month later).

The Da Vinci Xi™ platform provides a less invasive surgical approach for this method of hybrid ablation. Robotic-assisted approaches are rapidly increasing across various surgical disciplines, including cardiac surgery, as they offer an unrivaled degree of maneuverability with three-dimensional high-definition visualization. Seven degrees of freedom while maneuvering a range of surgical instruments not only mimics a surgeon’s natural manual abilities but furthers the surgeon’s range of movements. Fine dissection using robotic instruments is performed with greater ease than nonrobotic thoracoscopic approaches. Lysis of scar tissue using laparoscopic instruments is restrictive and may limit access to neurovascular and epicardial structures. Robotic dissection addresses this by allowing the surgeon to perform a more extensive and safe dissection in heavily scarred fields. Traditional approaches to the Convergent procedure also lack intraoperative electrophysiological mapping, necessitating greater numbers of lesions to achieve freedom from AF. Here, we describe the “Robotic Convergent Plus” procedure - a novel, robotic-assisted hybrid ablation for patients with persistent AF.

## Technical report

Preoperative brief and preparation

Preoperatively, the patient’s usual anticoagulation (nonvitamin K antagonist oral anticoagulants, NOACs, or coumadin) is continued up to the day of surgery and continued after for ninety days. Before the patient arrives in the OR, a multidisciplinary team preoperative briefing is undertaken. An institutional structure is followed for this briefing rather than just the standard WHO Surgical Safety Checklist, ensuring active involvement from all team members (Table 1).

**Table 1:** Surgical team members and contributions to the preoperative brief and preparation for surgery. Each member of the multidisciplinary surgical team has specific contributions to the pre-operative team briefing, as shown in this table.

Role	Contribution to Preoperative Brief
Responsible surgeon	Description of the patient’s history and indications for the procedure
Cardiovascular anesthesiologist	Description of preoperative transesophageal echocardiography (TEE) findings; Discussion of airway and anesthesia strategy
Scrub technicians	Special instrumentation requirements; Timing of key steps
OR nurses	Display of relevant imaging; Discussion of recent blood results; Setup of electrophysiological mapping equipment

In addition to standard instrumentation for robotic cardiac surgery via the Da Vinci Xi™ system, additional equipment is necessary for the Robotic Convergent Plus procedure to take place (Table 2). The normal operating function is verified for all surgical systems and the Da Vinci Xi™ robotic surgical system before use.

**Table 2:** Special equipment used in addition to standard instruments for robotic cardiac surgery with the Da Vinci Xi system. In addition to the standard instrumentation for robotic cardiac surgery using the Da Vinci Xi robotic surgical system, additional equipment is required for the epicardial ablations and left atrial appendage ligation, as detailed in this table.

System Name	Manufacturer	Function
EnSite Precision Cardiac Mapping™ system	Abbott Laboratories Inc., Chicago, IL	Electrophysiological mapping throughout the procedure
EPi-Sense ST™ ablation system, consisting of:	AtriCure Inc., Mason, OH	Epicardial ablations
1. A generator: either the RF generator or the Multifunctional Ablation Generator	AtriCure Inc., Mason, OH	Generate and transmit radiofrequency (RF) energy to the electrode of the ablation probe
2. EPi-Sense ST Ablation probe	AtriCure Inc., Mason, OH	Ablate epicardial structures
AtriClip ProV™	AtriCure Inc., Mason, OH	Epicardial left atrial appendage ligation

Anesthesia and positioning

General anesthesia with a double-lumen endotracheal tube (DLETT) and a bronchial blocker (BB) is our preferred technique for lung isolation and single-lung ventilation. We find that compared to a single-lumen endotracheal tube (SLETT) with a BB, DLETT use decreases time to establish satisfactory endotracheal anesthesia, does not require flexible bronchoscopy, and offers a larger diameter lumen for passage of tools such as fiber optic bronchoscopes or suction catheters. The combination of a DLETT with a BB has been previously reported, specifically in cases where lung isolation may be inadequate with just a DLETT [10].

Anesthesia and adequate double lung ventilation are achieved with the patient in the supine position. Before repositioning the patient, a repeat TEE is carried out by the cardiovascular anesthesiologist to check for a thrombus in the LAA. If the TEE is ambiguous, Definity® contrast (Lantheus Inc., North Billerica, MA) is administered to improve image quality. Definity® (octafluoropropane) is a highly echogenic substance that improves ultrasound signal backscatter, opacifying the cardiac chambers and thus improving delineation of any potential thrombus within the LAA. If an intracardiac thrombus is seen on TEE, then surgery is aborted due to the considerable risk of fragmentation and embolic events. Once TEE confirms that no thrombus is present, the CIRCA S-CATH M™ esophageal temperature probe (CIRCA Scientific, Inc., Englewood, CO) is prepared for placement.

**Figure 1:**
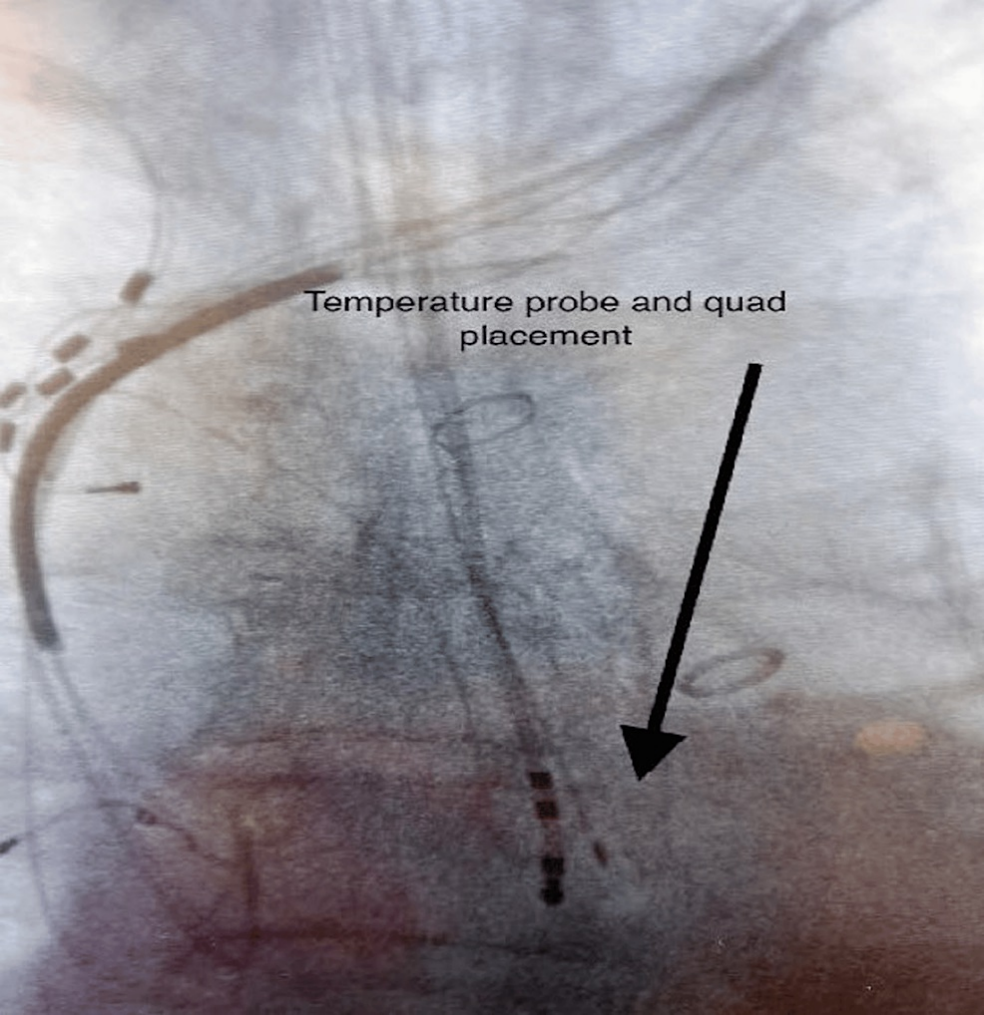
Placement of the CIRCA S-CATH M™ esophageal temperature probe under fluoroscopy. The placement of the esophageal temperature probe is confirmed using either fluoroscopy (as shown) or using transesophageal echocardiography (TEE). The position is usually confirmed using TEE in our clinical practice.

The distal ends of the quadrupole catheter (part of the Abbott ESI electrophysiological mapping system) and the esophageal temperature probe (CIRCA S-CATH) are sutured together, and the two are wrapped around each other. After that, both are orally placed in the esophagus by the anesthesiologist, alongside the TEE probe. The placement is typically such that the tip of the temperature probe and the quadrupole are one and a half to two vertebral bodies below the carina; the location is confirmed using TEE or fluoroscopy (Figure 1). An advantage to utilizing both a temperature probe and the quadrupole is two types of visualization of correct placement, with the quadrupole catheter visualized on the Abbott ESI 3D mapping system.

The patient is then positioned in the left lateral position with a pressure infuser bag placed under the left scapula. Later in the procedure, to allow optimal access for LAA ligation (LAAL), the left chest is elevated by inflating this pressure infuser bag. Arm protection is also placed to prevent excessive pressure at any point. After repositioning, the patient is prepped and draped in the usual fashion.

Monitoring and lines

Standard monitoring for cardiac surgery (TEE probe and telemetry as per American Society of Anesthesiologists (ASA) guidelines) are applied, as well as gel pads for electrocautery and cardioversion if necessary. The following vascular lines are placed in the typical sterile manner: Right IJ cordis, Swan-Ganz central line for flow dynamics monitoring, and right radial arterial line. Systemic unfractionated heparin is given intravenously to achieve a target-activated clotting time (ACT) of ≥ 480 seconds (300-400 IU/kg initial bolus, with additional boluses as necessary). Additionally, the esophageal temperature probe allows for luminal esophageal temperature (LET) monitoring, a key aspect of ensuring safe ablations.

Incisions and access

**Figure 2:**
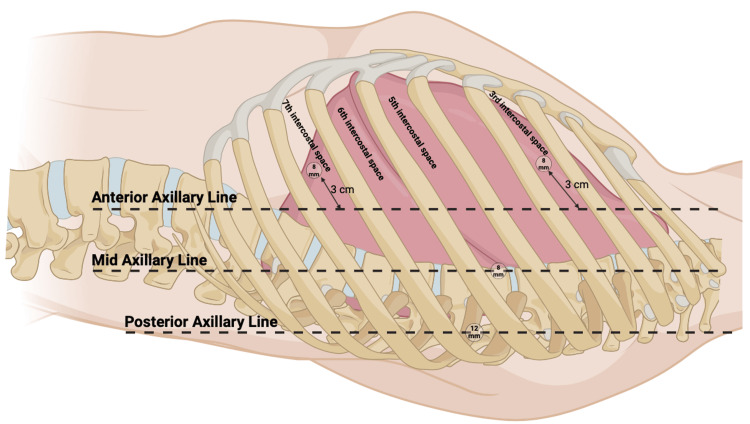
Incisions for port placement. Four incisions are made into the left chest for port placement – 3 cm medial to the anterior axillary line in the third and seventh intercostal spaces, at the level of the mid-axillary line in the fifth intercostal space, and at the level of the posterior axillary line in the sixth intercostal space. Figure created with BioRender.com [11]. Image Credits: BioRender original icons library.

Before an incision is made, a formal timeout is performed by the circulator in the room. The BB is then deployed, and the left lung is collapsed to achieve single-lung ventilation. Four incisions are made into the left chest wall for surgical access (Figure 2).

**Figure 3:**
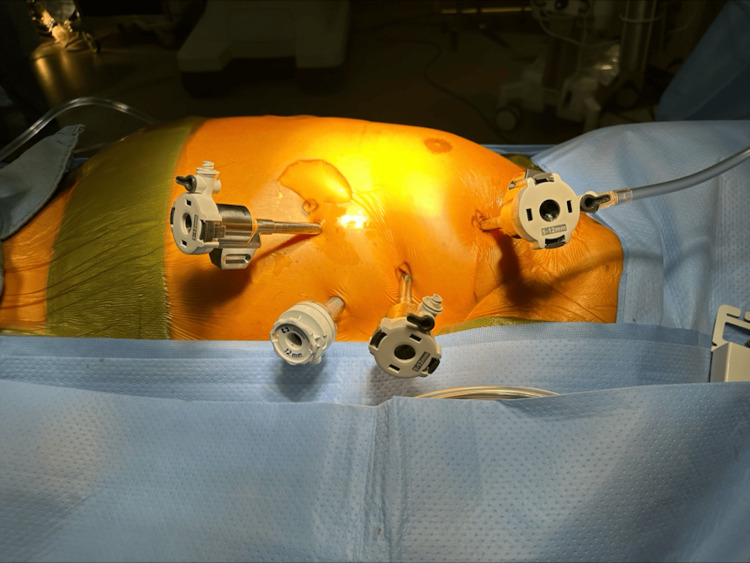
Port setup for robotic access. Port placement: 8 mm ports for the right and left robotic arms respectively in the third and seventh intercostal spaces, an 8-mm port for thoracoscopic visualization in the fifth intercostal space, and a 12-mm assistant’s working port in the sixth intercostal space.

A 12-millimeter working port is placed in the sixth intercostal space at the level of the posterior axillary line. Two eight-millimeter ports are then placed three centimeters medial to the anterior axillary line, at the level of the third and seventh intercostal spaces. Another eight-millimeter port is placed into the fifth intercostal space at the level of the mid-axillary line. The complete port setup is shown in Figure 3.

A 30-degree scope (Da Vinci Xi™ 8 mm Endoscope Plus, Intuitive Surgical, Sunnyvale, CA) is introduced through the third intercostal port and connected to the Da Vinci Vision Cart. The Da Vinci Xi™ system is then brought in from the left side and the thorax is selected on the robot touchscreen console before arm deployment for docking. We do not flex the bed as the increase in intercostal space is limited and of little use at the thoracic apex. This streamlines the operative procedure, reducing anesthesia time. One of the great advantages of the Da Vinci Xi™ compared to other robotic surgical systems is the wide range of port placements that can be used without compromising functionality.

Primarily, the third intercostal port is used for the right robotic arm, the seventh intercostal port is used for the left robotic arm, and the fifth intercostal port is used for thoracoscopic visualization via the third robotic arm. While we perform this procedure with three arms, a fourth arm would provide a means of additional retraction, particularly in cardiomyopathy patients with large hearts. However, given that the assistant’s working port is needed for the introduction and manipulation of the electrophysiological mapping and RFA probes as well as for the introduction of the epicardial LAA clip, we tend not to use a fourth arm, utilizing the assistant for additional retraction if needed.

Through the ports, robotic instruments are introduced (Cadiere forceps, cardiac grasper, and monopolar curved scissors: Intuitive Surgical, Sunnyvale, CA), and mediastinal soft tissue is dissected to reach and identify the pericardium. Next, the caudal aspect of the anterior pericardium is incised to create a horizontal pericardiotomy of 3-4 cm in length, and the phrenic nerve is identified. Pericardial fluid is aspirated to confirm entry into the pericardial space and removed to enhance visualization.

Epicardial mapping using the Abbott EnSite Precision™ mapping system

**Figure 4:**
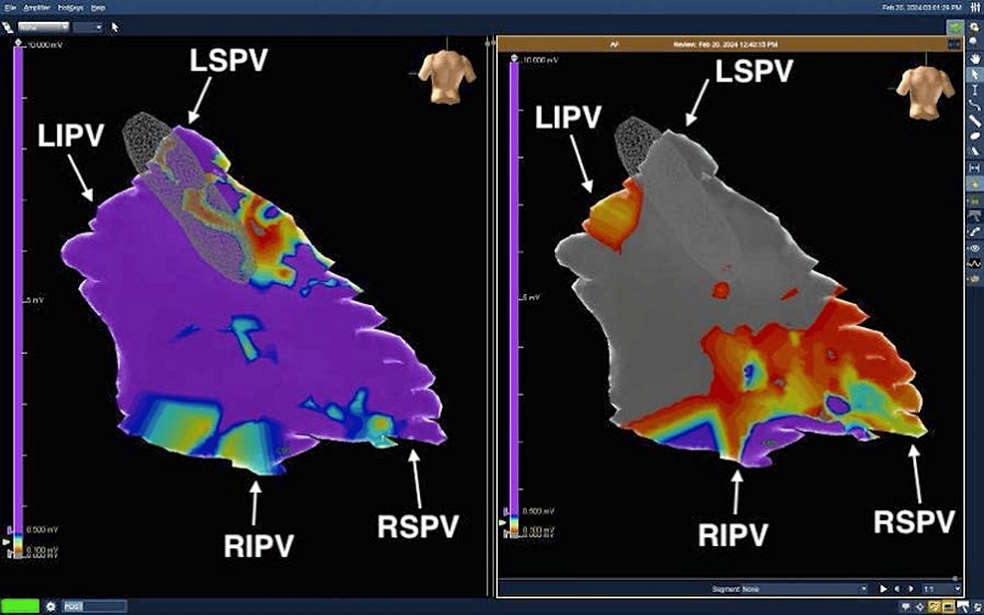
Electrophysiological mapping before (left) and after (right) epicardial ablation: significantly reduced electrical activity following ablation. The left atrial posterior epicardial wall is shown here, with the four pulmonary veins marked: the LSPV is at 11 o’clock, the LIPV is at 9 o'clock, the RSPV is at 4 o'clock and the RIPV is at 6 o'clock. A significant reduction in electrical activity can be seen in the right panel (post-ablation) compared to the left panel (pre-ablation), with some residual activity around the entrances of the RSPV, the RIPV, and the LIPV. These would subsequently be "touched up" in the follow-up visit to the EP, who would usually perform a complete pulmonary vein isolation ablation using a catheter-based approach, as well as any other sites of residual electrical activity.

First, the Abbott EnSite Precision™ mapping grid probe is introduced via the working port. The Abbott ESI™ software renders a three-dimensional electrophysiological map using readings from the probe as it is moved in a path across the left atrial epicardial surface. This path starts by mapping the left and right pulmonary veins as they enter the left atrium, proceeding to map the posterior wall and roof line of the left atrium, and the coumadin ridge. Residual AF signals after subsequent ablations are sensed using voltage maps from esophageal electrocardiogram EKG recordings. These voltage maps serve as a surrogate for post-ablation tissue viability, ensuring complete electrical silence and confirming the eradication of AF substrates (Figure 4).

Ablations

Once electrophysiological mapping is completed, a zero-degree robotic thoracoscope (Da Vinci Xi™ 8 mm Endoscope Plus, Intuitive Surgical, Sunnyvale, CA) is introduced through the 12-millimeter working port. A fully collapsed left lung is confirmed and key landmarks are identified, including the ligament of Marshall (LOM), coronary sinus, left inferior pulmonary vein, and right inferior pulmonary vein. The scope is then withdrawn and reintroduced through the fifth intercostal port. Next, the EPi-Sense ST™ Coagulation Device (AtriCure Inc., Mason, OH) monopolar RFA catheter is introduced through the working port.

**Figure 5:**
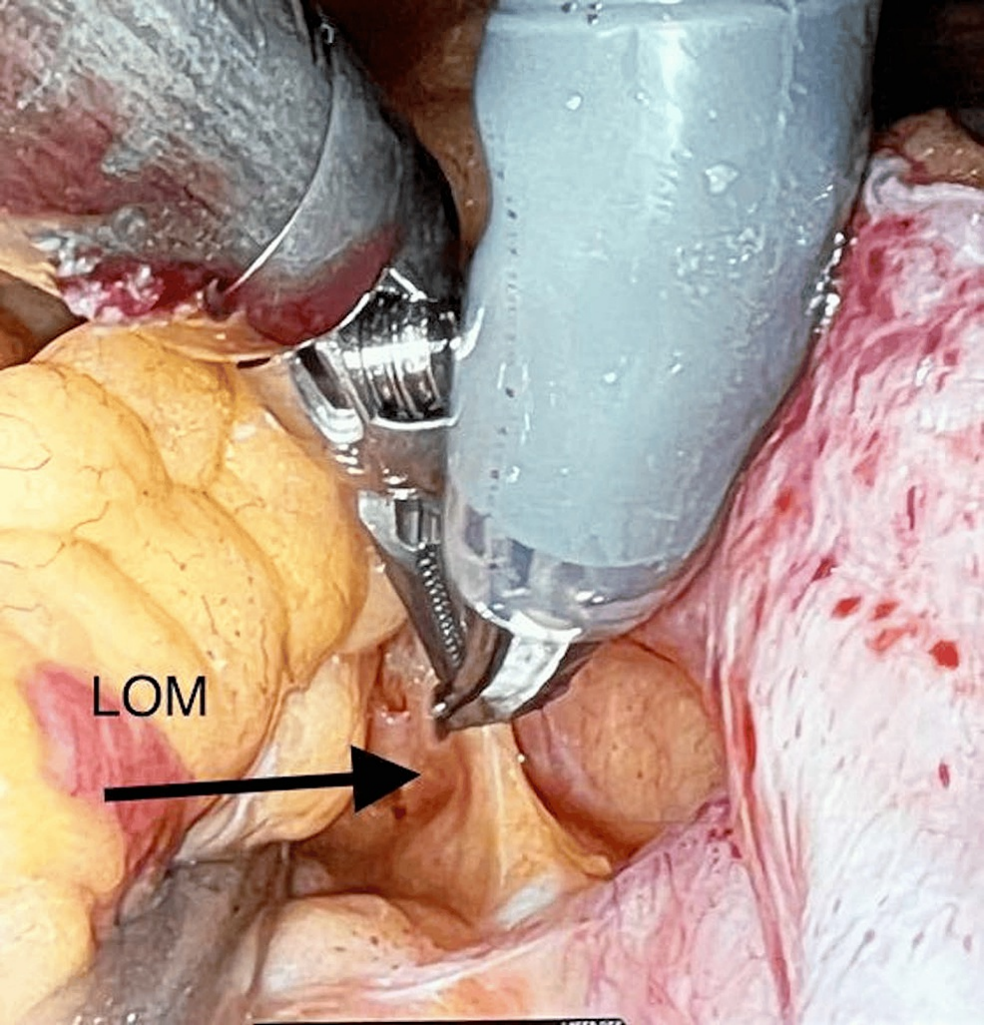
Ablation of the ligament of Marshall The ligament of Marshall is divided and ablated using monopolar curved scissors (Intuitive Surgical, Sunnyvale, CA).

**Figure 6:**
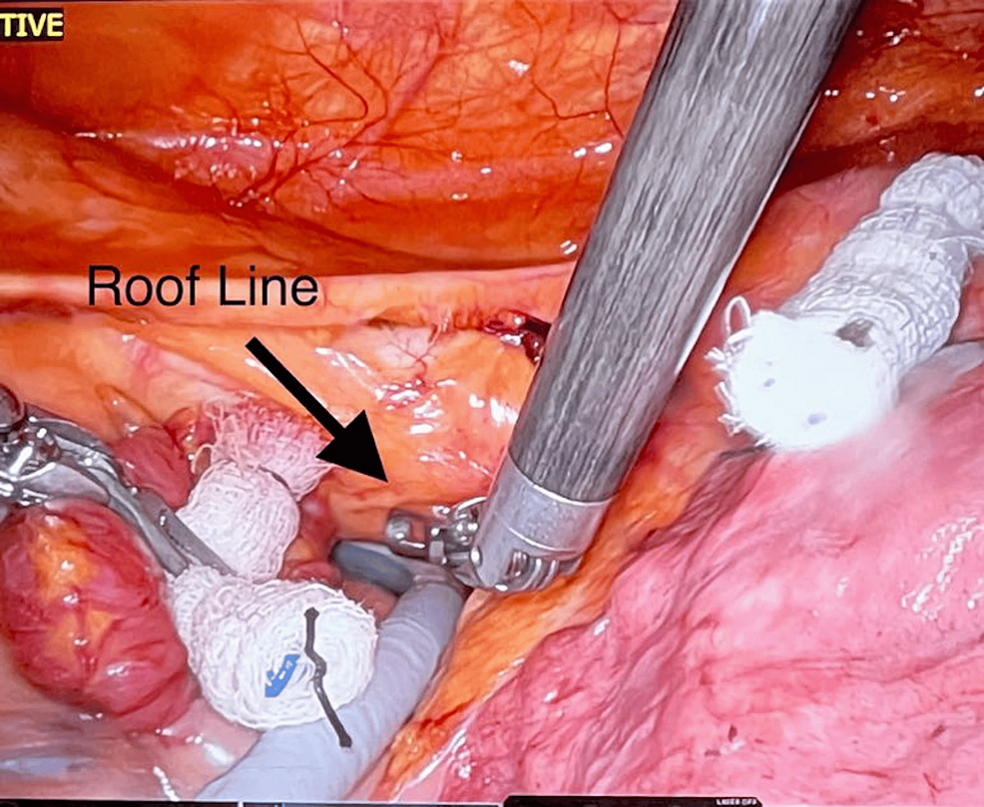
Ablation of the roof line Ablations are made along the roofline of the left atrium using the EPi-Sense ST ablation probe (AtriCure Inc., Mason, OH).

**Figure 7:**
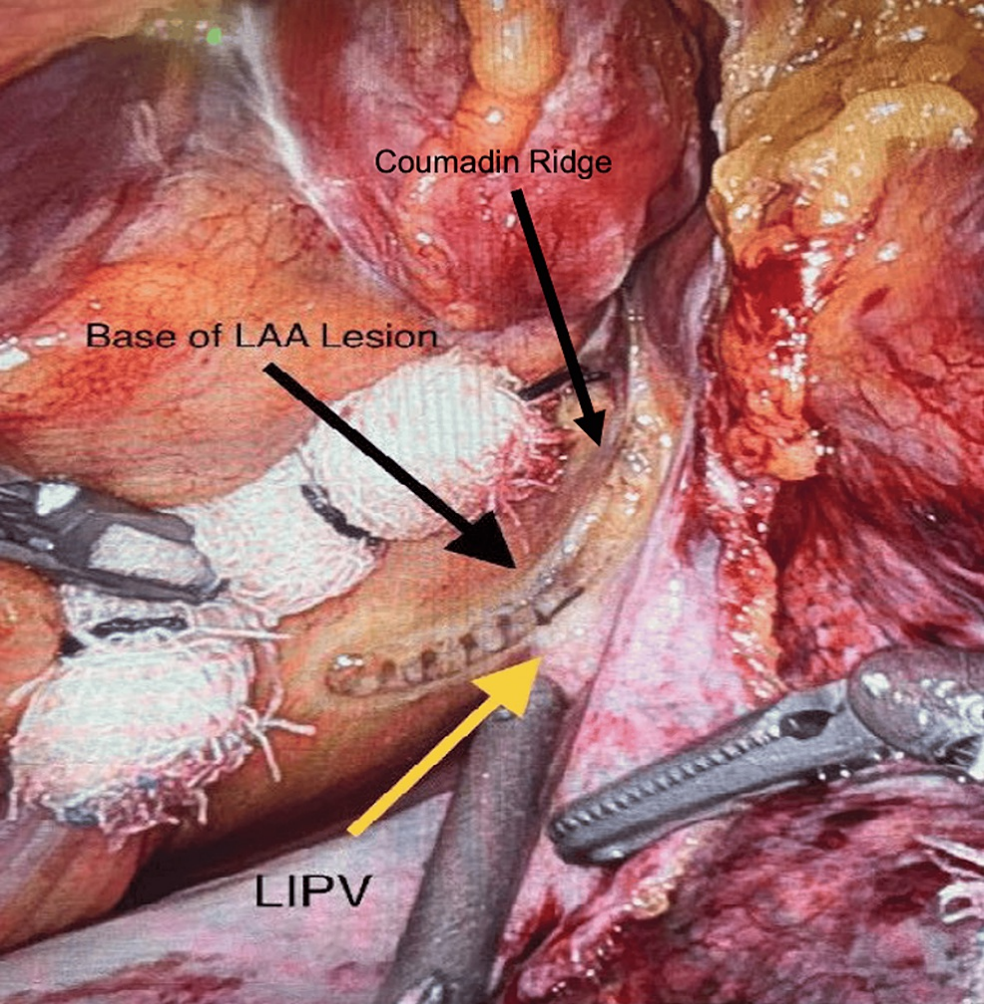
Ablation of the base of the left atrial appendage and a line from the coumadin ridge to the left inferior pulmonary vein. Ablations are made in a line from the coumadin ridge to the left inferior pulmonary vein using the EPi-Sense ST ablation probe.

**Figure 8:**
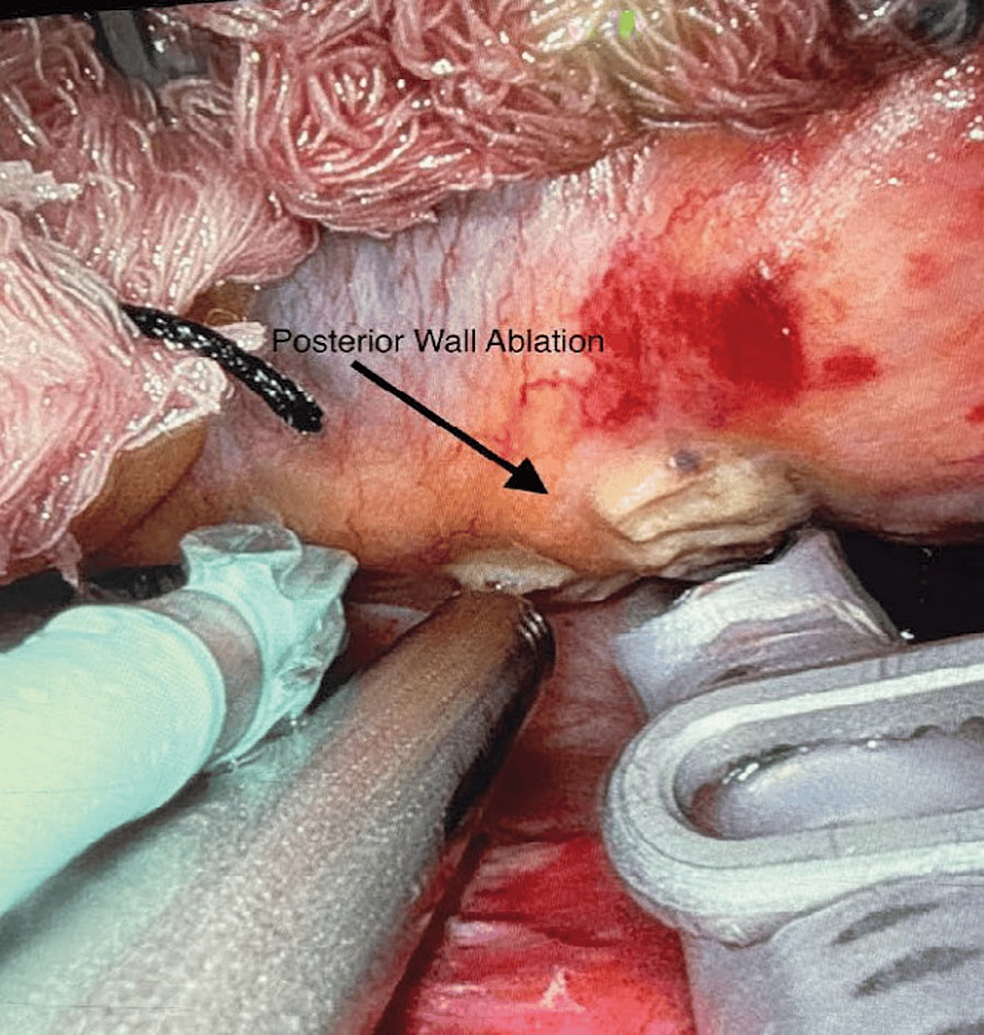
Ablation of the posterior wall of the left atrium. Ablations are made along the posterior wall of the left atrium using the EPi-Sense ST ablation probe.

First, using the monopolar curved scissors, the LOM is ablated and divided (Figure 5). A series of transmural ablation lesions are created using the EPi-Sense ST™ RFA probe at the roofline of the left atrium, the base of the LAA, and a line from the coumadin ridge to the left inferior pulmonary vein (Figures 6, 7). Finally, the posterior left atrial wall is ablated (Figure 8). The RF power is preset on the generator at 30 watts, as is the amount of time for each application of the ablation probe and subsequent ablation (90 seconds). The Epi-Sense ST ablation probe is impedance-based - the lower the impedance, the higher the power.

The EPi-Sense ST™ device detects an electromyogram before and after each ablation. This assessment of the electrical signal is essential to confirm electrical silence at the lesion site and is documented after every burn. Confirming electrical silence at targeted sites increases procedural success and minimizes the total number of lesions, thereby minimizing unnecessary myocardial scar tissue formation.

Our institution utilizes continuous LET monitoring using the CIRCA S-CATH M™ probe. If the temperature sensed by this probe during each ablation increases by greater than 0.5°C compared to before starting the ablation, then the burn is aborted. This low threshold for interruption of energy delivery is necessary to minimize the risk of esophageal injury caused by excessive LET rises.

Left atrial appendage ligation

**Figure 9:**
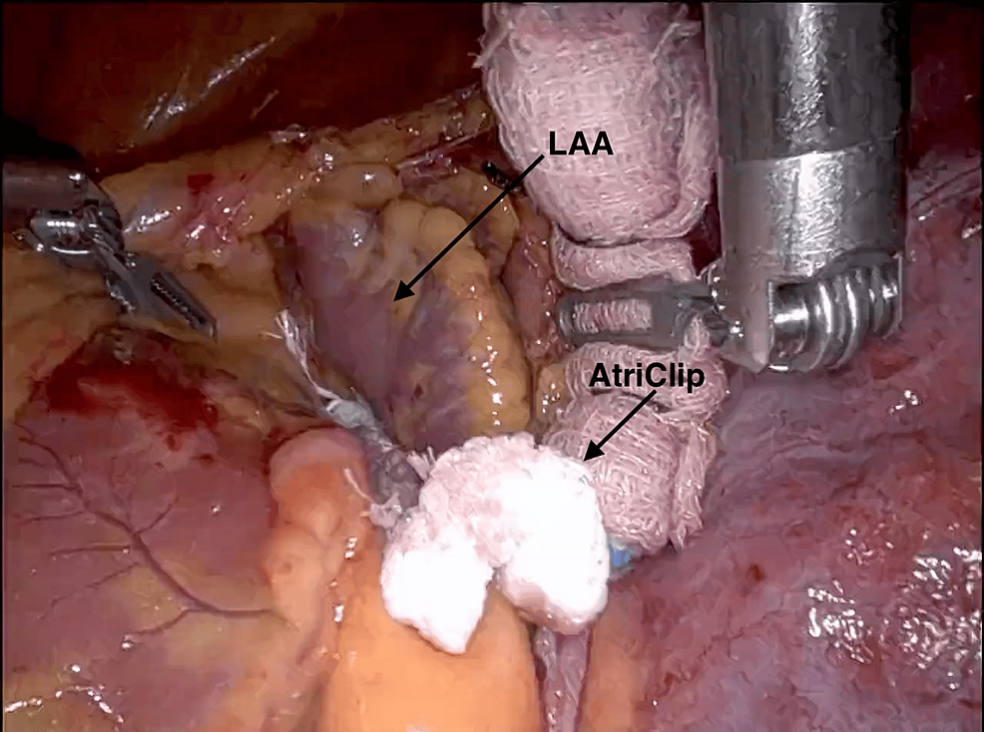
Placement of the AtriClip ProV device for left atrial appendage ligation. The left atrial appendage is completely ligated using an AtriClip ProV device (AtriCure Inc., Mason, OH). The anesthesiologist and surgeon confirm that < 0.3 cm stump remains using TEE with at least three angles.

Once epicardial ablation is completed, attention is turned to LAAL. The anesthesiologist once again confirms via TEE that there is no intracardiac thrombus, with Definity® contrast administered in cases of ambiguity. The pressure infuser bag which had been placed under the left scapula is now inflated to raise the left hemithorax. Using robotic instruments, the phrenic nerve is identified, and mediastinal fat is retracted anteriorly. The AtriClip™ Selection Guide (AtriCure, Mason, OH) is introduced via the working port to measure the LAA and select an appropriate size for the AtriClip ProV™ device. The chosen LAA clip is introduced via the working port and directed around the appendage base. A rolled-up radio-opaque surgical sponge and robotic instruments are used to retract the LAA and facilitate the appropriate positioning of the clip. The clip is then closed but not locked or released from the deployment handle. Using TEE, the anesthesiologist confirms appropriate clip placement, ensuring that ≤ 0.3 cm of stump is visible. The operating surgeon and anesthesiologist review at least three angles to ensure the minimum residual stump remains. Once optimal clip placement is confirmed, the device is locked and released from the handle (Figure 9).

Closure

All four robotic ports are removed under direct visualization. For postoperative drainage, one chest tube (24Fr Blake connected to a JP bulb - ClearFlow Inc., Irvine, CA) is placed through the 7th intercostal space incision and secured in the usual manner. The EKG trace is inspected - if the patient is still in AF, they are cardioverted to return to normal sinus rhythm. The BB is collapsed, and the left lung is reinflated under direct visualization to ensure good apposition of the upper, anterior, medial, and lateral lung surfaces to the rib cage. A standard sponge, needle, and instrument count is conducted before the closure of the surgical wounds. Local anesthesia is infiltrated around each of the four incisions before the subcutaneous tissues are approximated in layers using 0 and 2-0 Stratafix™ and the skin is closed with one layer of subcuticular 4-0 Stratafix™ and Dermabond Prineo™. The patient is extubated before leaving the operating room.

Postoperative care and follow-up

Upon arrival in the intensive care unit (ICU), a multi-disciplinary handoff is performed, and a postoperative chest x-ray is reviewed by the team. The chest tube is removed once output is less than 150 mL over 24 hours. Postoperatively, anticoagulation is continued for ninety days, providing adequate thromboprophylaxis until freedom from AF can be confirmed. No postoperative driving restrictions are necessary, with minimal opiates to be used.

Patients are followed up at the standard intervals of two weeks postoperatively, eight weeks, and six months by the cardiac surgical team. The patient follows up with their electrophysiologist after 30 days for the endocardial aspect of the convergent procedure. At this stage, electrophysiological mapping is once again carried out and compared with pre-operative electrical activity. The patient is also followed up by the electrophysiologist at the two-week, eight-week, and six-month intervals following catheter ablation, corresponding to eight weeks, three months/90 days, and seven months following the epicardial ablation. The ongoing need for anticoagulation is reviewed at 90 days follow-up with the EP as part of the “arrhythmia multidisciplinary team”. Holter monitoring is also undertaken to verify freedom from AF - this, along with clinical assessment, guides cessation of anticoagulation and anti-arrhythmic medications, with further electrophysiological mapping being unnecessary.

## Discussion

We aimed to evaluate the feasibility of a novel robotic approach to hybrid ablation in patients with persistent AF. In developing the “Robotic Convergent Plus” procedure, several modifications were made to the conventional technique for the Convergent procedure, including intraoperative electrophysiological mapping and continuous LETM. The integration of three-dimensional electrophysiological mapping technologies allows the surgeon to operate with greater precision. Notably, the elevation of the ablation catheter off the posterior surface of the pericardium minimizes heat dispersion, thereby preserving the integrity of surrounding tissues and optimizing therapeutic outcomes, while mitigating LET rises, which is further enhanced by continuous LETM [12].

While esophageal thermal injury risk is lower with epicardial ablation compared to endocardial approaches, there is published evidence of lethal iatrogenic atrio-esophageal fistulae following epicardial ablation [13]. It is conventional to empirically limit the RF power to avoid esophageal thermal injury, particularly when ablating the posterior left atrial wall. Continuous LETM using the CIRCA S-CATH M™ probe allows the safe use of a higher power when creating lesions while minimizing the risk of thermal esophageal injury. This method has previously been described to reduce lesion sets [14,15]. Notably, one of the advantages of the robot is that we usually lift the posterior wall with the left robotic arm and use the cardiac grasper to lift the EPi-Sense probe off the back of the pericardial wall further limiting contact and transfer of heat. During an ablation, we irrigate with cool sterile saline. Additionally, we wrap the temperature probe with the ESI mapping system quadrupole lead so that in real-time during the ablation, we have a direct line of sight of the location of the temperature probe in relation to the ablation catheter, making for a safer burn. Lastly, at the end of each burn, we also inject 200mg (50mL) of Kenalog (Triamcinolone) topically at the back of the left atrium to minimize inflammation. The success of this approach is further demonstrated in our institution’s lack of esophageal injuries.

With our significant institutional experience of surgical ablation for persistent AF, we find our totally robotic approach to the Convergent procedure to offer a variety of advantages over a non-robotic thoracoscopic subxiphoid Convergent procedure. Using the approach as described in this manuscript, our team has successfully performed 38 totally robotic convergent procedures. All patients were discharged without complication, thus validating the safety and feasibility of this approach. A comprehensive analysis of outcomes is soon to be shared in future work from our group once all patients have undergone the catheter-based ablation, to assess AF burden and freedom from anti-arrhythmic medications.

Robotic convergent vs subxiphoid access

While other robotic surgical systems exist, the Da Vinci Xi™ system is the most advanced and ubiquitous system available to surgeons. It is the system on which the greatest number of surgical staff are trained and experienced. With its wide range of instruments and high-definition three-dimensional visualization system, the Da Vinci Xi™ system provides a far superior view when compared to the traditional thoracoscopic approach. Moreover, the Da Vinci Xi™ system facilitates superior visualization, enabling the identification and targeting of critical anatomical structures such as the ligament of Marshall, the roofline, the posterior left atrium, and lines from the Coumadin ridge to the left inferior pulmonary vein. This heightened visual acuity is further augmented by the utilization of the EPi-Sense ST™ steerable catheter, which enables precise maneuverability and mapping within the epicardial space.

The Da Vinci Xi™ robotic system has seven degrees of instrument freedom, which facilitates the dissection of adhesions and anatomical structures with enhanced comfort and control. This maneuverability minimizes the risk of operative trauma and complications [16]. One additional notable advantage of employing the Da Vinci Xi™ robotic system lies in its procedural efficiency when compared with the subxiphoid approach. Comparisons of our institutional experience with both approaches have shown that utilizing this robotic platform reduces the average procedural duration by 30-60 minutes, thereby minimizing operative time and associated risks. The typical operative time of this approach is between 1.5 and 2 hours for 15-20 ablations, typically two on the roofline, two on the coumadin ridge, one connecting from the coumadin ridge to the left inferior pulmonary vein, and 10-15 on the posterior left atrium depending on the size of the left atrium. Comparatively, the typical procedural time via a subxiphoid approach with robotic LAAL is around 3.5 hours. This decrease in procedural time is attributable to the fact that one robotic setup and docking allows the entire procedure to be carried out, rather than setting up a subxiphoid approach for ablations and mapping, before repositioning and setting up for robotic LAAL. The enhanced visualization and better access to key structures additionally improve operative time, reducing the amount of time spent maneuvering instruments and the RFA system.

Compared with the subxiphoid approach, the robotic approach provides greater patient satisfaction and a better postoperative experience for patients in several ways. There is significantly less postoperative pain, better cosmetic appearance of surgical wounds, and shorter postoperative length of stay. Previously, patients were not permitted to drive for two weeks postoperatively to protect the subxiphoid incision from incidental airbag deployment. Since robotic ports are placed on the lateral chest wall, this driving restriction is not necessary with the robotic approach.

Outcomes and potential complications

Lastly, due to the less invasive approach than the subxiphoid approach, as well as meticulous intraoperative hemostasis, we have had excellent outcomes with postoperative recovery, with no patients experiencing significant postoperative bleeding or requiring re-operation.

Limitations

While the Robotic Convergent Plus procedure is a valid approach for patients with persistent AF, it is not suitable for patients requiring other cardiac surgical procedures such as valve surgery. For such patients, the Maze procedure is ideal as it can be performed concomitantly, making use of the sternotomy or thoracotomy access and CPB setup being used for the other cardiac surgery.

In patients with particularly large hearts with cardiomyopathy, our three-arm approach may provide sub-optimal exposure to the key structures. While some may find it beneficial to use a fourth arm to help with access and visualization, our experience showed the fourth arm often obstructs the movement of the other arms as well as hinders effective access to structures for mapping and ablation. Additionally, the fourth arm can lead to more arm conflicts than the benefit the additional retraction provides. Our institutional practice is therefore for the bedside assistant to provide additional retraction via the working port; however, this may still be sub-optimal in patients with particularly large hearts.

## Conclusions

The Da Vinci Xi™ robotic surgical system provides a safe, precise, reproducible, and improved approach to epicardial ablation for persistent AF. Through increased range of movement and improved access to key structures such as the coumadin ridge and left atrial roofline, we are increasingly successful in providing patients with long-term freedom from AF, improving both symptoms and prognosis. Also, implementing three-dimensional epicardial electrophysiological mapping throughout the procedure, and before and after each set of ablations confers additional precision and intelligence to our ablation strategy. We can more accurately target areas of disorganized electrical activity and verify electrical silencing following ablation, minimizing the number of “unnecessary” ablations.

Compared to traditional methods of surgical ablation such as the Cox-Maze IV (“Maze” procedure), a hybrid approach offers significant advantages. The Maze procedure requires CPB and sternotomy, which are associated with higher morbidity and mortality than catheter-based ablations. Variations such as the “Mini-Maze” achieve similar lesion sets without the need for a median sternotomy. The Mini-Maze can be unilateral or bilateral, not necessarily requiring CPB, though presenting significant anatomical challenges, with multiple incisions required to achieve suitable access to key structures. This results in significant postoperative pain and the potential for wound complications.

Compared to the non-robotic subxiphoid approach previously used, there is much less postoperative pain, with better cosmetic appearance and faster wound recovery. Previously patients remained in hospital for two days postoperatively rather than one in this procedure and were not permitted to drive for two weeks postoperatively to protect the incisions in case of airbag deployment, whereas this is no longer a concern with the totally robotic approach. The use of the Da Vinci Xi™ robotic surgical system provides a superior approach for both patient and surgeon.
